# A WNT7B-m^6^A-TCF7L2 positive feedback loop promotes gastric cancer progression and metastasis

**DOI:** 10.1038/s41392-020-00397-z

**Published:** 2021-02-02

**Authors:** Qian Gao, Liuyang Yang, Aolin Shen, Yang Li, Yongxiang Li, Shilian Hu, Runhuai Yang, Xiangting Wang, Xuebiao Yao, Guodong Shen

**Affiliations:** 1 Department of Geriatrics, The First Affiliated Hospital of USTC, Division of Life Sciences and Medicine, University of Science and Technology of China, Hefei, Anhui 230001 China; 2 Department of Genetics, School of Life Science, Anhui Medical University, Hefei, Anhui 230032 China; 3 Anhui Provincial Key Laboratory of Tumor Immunotherapy and Nutrition Therapy, Hefei, Anhui 230001 China; 4 Department of General Surgery, First Affiliated Hospital of Anhui Medical University, Hefei, Anhui 230022 China; 5 School of Biomedical Engineering, Anhui Medical University, Hefei, Anhui 230032 China; 6 MOE Key Laboratory for Membrane-less Organelles and Cellular Dynamics, University of Science and Technology of China, Hefei, Anhui 230027 China

**Keywords:** Metastasis, Gastrointestinal cancer, Epigenetics

**Dear Editor,**

Signal transduction takes the responsibility of translating extracellular information into specific cellular activities, enabling cells, including cancer cells, to respond exquisitely to extracellular guidance cues. N^6^-methyladenosine (m^6^A), the most pervasive and abundant modification within eukaryotic mRNAs, is known to have specific effects on cellular activities relevant to cancer.^1^ Despite the fact that m^6^A methylation is a dynamic event involving a series of enzymes, it remains unknown whether signal transduction, much less extracellular signaling molecules, uses m^6^A methylation as an effector mechanism in cancer.

Gastric cancer carries a poor prognosis, mainly due to its high proclivity to metastasize and the lack of bona fide biomarkers for early diagnosis and precision-targeted therapy. In addition to upregulated m^6^A methylation,^2^ gastric cancer harbors hyper-activated Wnt/β-catenin signaling, whose underlying mechanism is unclear.^3^ Given that cross talk with other mechanisms often influences Wnt/β-catenin signaling activation, here we investigated whether Wnt/β-catenin signaling synergized with m^6^A methylation in gastric cancer.

Transcription Factor 7 like 2 (TCF7L2), a core component binding to nuclear β-catenin to transduce Wnt signaling,^4^ was upregulated in gastric cancer tissues (Fig. 1a). High TCF7L2 expression was associated with aggressive clinical features, poor overall survival, and high recurrence rate of patients (Supplmentary Fig. S1). Subsequently, TCF7L2 was either knocked down by shRNAs in N87 and 44As3 cells or overexpressed in SGC7901 cells to evaluate the correlation between TCF7L2 expression and malignant phenotypes of gastric cancer cells (Supplmentary Fig. S2). Our results showed that TCF7L2 endowed gastric cancer cells with the advantage of proliferation and migration, and induced the gastric cancer stem cell phenotype by strengthening the capacity for self-renewal and upregulating the expression of stem cell markers. The notable role of TCF7L2 in promoting gastric cancer growth and metastasis has also been demonstrated in heterotopic and orthotopic mouse models. Accordingly, TCF7L2 could promote metastatic progression of gastric cancer.

**Fig. 1:**
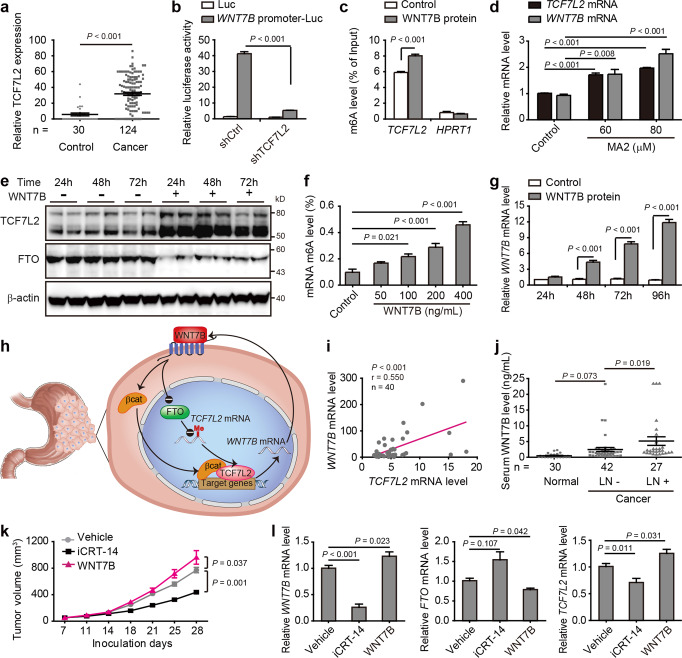
The role of the WNT7B-m6A-TCF7L2 positive feedback loop in gastric cancer metastatic progression. **a** Immunohistochemical staining of TCF7L2 protein in gastric tissues from patients with gastric cancer (Cancer) and chronic gastritis (Control) was quantitatively measured using Image J software. **b** Luciferase reporter assay for *WNT7B* promoter in N87 cells transfected with TCF7L2 shRNA. **c** m^6^A methylation of *TCF7L2* mRNA in N87 cells treated with WNT7B protein (200 ng/mL) for 48 h was analyzed by gene-specific m^6^A-RIP-qPCR. *HPRT1* served as a negative control. **d** mRNA levels of *TCF7L2* and *WNT7B* in N87 cells treated with MA2 for 48 h were analyzed by qRT-PCR. **e** Expression of TCF7L2 and FTO at the indicated time points (two biological replicates for each) was analyzed by immunoblotting in N87 cells treated with WNT7B protein (200 ng/mL). **f** ELISA-based assay for quantification of global m^6^A levels in N87 cells treated with WNT7B for 48 h. **g** Levels of *WNT7B* mRNA at indicated time points were analyzed by qRT-PCR in N87 cells treated with WNT7B protein (200 ng/mL). **h** Proposed working model of the WNT7B-m^6^A-TCF7L2 positive feedback loop. **i** Correlation between *WNT7B* and *TCF7L2* mRNA levels in cancer tissues from patients with gastric cancer. **j** Levels of WNT7B protein in the serum of healthy people undergoing health check-ups (Normal), gastric cancer patients without lymph node metastasis (LN−), and those with lymph node metastasis (LN+) were analyzed by ELISA. **k** PDX tumor growth curves for vehicle-, iCRT-14-, and WNT7B-treated mice are shown. *N*=6 mice per group. **l** mRNA levels of *WNT7B*, *FTO*, and *TCF7L2* in PDX tumors from vehicle-, iCRT-14-, and WNT7B-treated mice were analyzed by qRT-PCR. *P* values were calculated with the unpaired Student *t* test (**a**-**c**, **g**), one-way ANOVA with post hoc LSD test (**d**, **f**, **j**, **l**), Pearson’s correlation test (**i**), or two-way repeated-measure ANOVA with post hoc LSD test (**k**)

To probe the mechanisms underlying TCF7L2’s essential role in gastric cancer metastasis, we performed KEGG pathway enrichment analysis of differentially expressed proteins in TCF7L2-depleted N87 cells, and found that Wnt/β-catenin signaling was the most significantly enriched pathway (Supplmentary Fig. S3a). Furthermore, TCF7L2 utilized the oncogenic target genes of Wnt/β-catenin signaling to exacerbate gastric cancer (Supplmentary Fig. S3b–f). Interestingly, among Wnt signaling-related genes that encode secreted proteins, *WNT7B* had the largest expression change during modulation of TCF7L2 expression (Supplmentary Table S1, S2, and Supplmentary Fig. S4a), implying that its expression was under tight regulation of TCF7L2, which has not been reported so far. This was further validated by the results showing that both *WNT7B* mRNA levels of gastric cancer cells and WNT7B protein-released amount in gastric cancer cell culture medium were positively correlated with TCF7L2 expression (Supplmentary Fig. S4b, c). Furthermore, with primer sets covering the putative binding sites provided by the JASPAR database, ChIP analysis revealed that TCF7L2 directly bound to the region (−1777 to −1668) of the *WNT7B* promoter (Supplmentary Fig. S4d). TCF7L2 knockdown attenuated *WNT7B* promoter activity in N87 (Fig. 1b) and 44As3 cells (Supplmentary Fig. S4e). Disruption of TCF7L2/β-catenin transcriptional activity by iCRT-14 dose-dependently reduced WNT7B abundance (Supplmentary Fig. S4f). Moreover, WNT7B resembled TCF7L2 in enhancing the metastatic properties of gastric cancer cells (Supplmentary Fig. S4g–j). Together, our results identify TCF7L2 as a transcriptional activator of *WNT7B* in gastric cancer cells.

m^6^A methylation has been shown to play a critical role in regulating gene expression,^1^ while gastric cancer displays elevated m^6^A levels.^2^ The SRAMP online tool predicted that five m^6^A sites with high confidence were located in the UTR of *TCF7L2* mRNA (Supplmentary Fig. S5a). Thus, we analyzed the m^6^A methylation of *TCF7L2* mRNA to explore the mechanism underlying TCF7L2 upregulation. A noticeable enrichment in m^6^A levels of *TCF7L2* mRNA was detected in 3’UTR close to the stop codon (Supplmentary Fig. S5b), while the levels were even higher in WNT7B-stimulated cells (Fig. 1c). The dynamic m^6^A methylation of *TCF7L2* mRNA was further validated by m^6^A level changes induced by modulating the expression of m^6^A methyltransferase METTL3 and inhibiting FTO, which was the first identified demethylase of m^6^A modification (Supplmentary Fig. S6c, d). Furthermore, METTL3 knockdown decreased *TCF7L2* mRNA levels (Supplmentary Fig. S5e). Inhibiting FTO-mediated demethylation by MA2 increased *TCF7L2* mRNA levels along with *WNT7B* mRNA levels (Fig. 1d), indicating that m^6^A methylation positively regulates TCF7L2 expression. Consistently, the *TCF7L2* mRNA (Supplmentary Fig. S5f) and protein (Fig. 1e) levels of WNT7B-stimulated cells dramatically increased and remained elevated throughout the entire stimulation period (96 h). To our surprise, the mRNA (Supplmentary Fig. S5f) and protein (Fig. 1e) levels of FTO exhibited an opposite trend in changes upon WNT7B stimulation, revealing a novel role for WNT7B in negatively regulating FTO. WNT7B stimulation could not obviously change the mRNA levels of *METTL3*, which has been implicated in gastric cancer progression (Supplmentary Fig. S5f).^5^ Nevertheless, global m^6^A levels in mRNA were remarkably elevated by WNT7B (Fig. 1f). Therefore, by downregulating FTO and thereby enhancing m^6^A methylation, WNT7B increased TCF7L2 expression in gastric cancer cells.

In line with the previous report that WNT7B was involved in Wnt/β-catenin signaling activation,^4^ WNT7B stimulation elevated nuclear β-catenin levels in gastric cancer cells (Fig. S5g). With upregulation of TCF7L2 abundance and nuclear translocation of β-catenin, WNT7B effectively activated the transcriptional activity of TCF7L2/β-catenin (Supplmentary Fig. S5h). Accordingly, there was a reciprocal regulatory loop between WNT7B and TCF7L2. In this setting, treating N87 cells with WNT7B protein induced a continuous increase in *WNT7B* mRNA during the 96-h period examined (Fig. 1g). Collectively, our findings reveal a WNT7B-m^6^A-TCF7L2 positive feedback loop wherein TCF7L2 enhances WNT7B expression by transducing Wnt/β-catenin signaling and WNT7B elevates TCF7L2 abundance via upregulating m^6^A methylation (Fig. 1h).

The significance of the positive feedback loop was further investigated in clinical samples. Consistent with the observations in gastric cancer cells, FTO expression was reduced in gastric cancer tissues (Supplmentary Fig. S6a), whereas WNT7B expression was upregulated and positively correlated with the recurrence rate (Supplmentary Fig. S6b–d), which was similar to the TCF7L2 expression pattern. More importantly, a positive correlation between *WNT7B* and *TCF7L2* mRNA levels was manifested in gastric cancer tissues (Fig. 1i). As shown in Fig. 1j, a progressive increase in serum WNT7B levels between healthy people, gastric cancer patients without lymph node metastasis, and those with lymph node metastasis indicated that serum WNT7B was positively correlated with metastatic progression (*r* = 0.646, *P* < 0.001, Spearman’s Rho), potentially facilitating noninvasive diagnosis and monitoring of gastric cancer.

To evaluate the therapeutic potential of targeting the positive feedback loop, we established patient-derived xenograft (PDX) models of gastric cancer. Tumor fragments from one patient with gastric cancer were implanted subcutaneously and treated via intratumoral injection with WNT7B, iCRT-14, or vehicle twice a week. Tumor growth was effectively suppressed by iCRT-14, but promoted by WNT7B (Fig. 1k and Supplmentary Fig. S6e, f). Moreover, iCRT-14 treatment resulted in a significant reduction in mRNA levels of *WNT7B* and *TCF7L2* but an increase in *FTO* mRNA levels in PDX tumors, whereas WNT7B treatment had the opposite effect (Fig. 1l), consistent with our in vitro data. METTL3 expression remained unchanged (Supplmentary Fig. S6g). These results collectively suggest that targeting the WNT7B-m^6^A-TCF7L2 positive feedback loop suppresses gastric cancer in vivo, which may serve as a novel therapeutic strategy.

In summary, our study identifies a WNT7B-m^6^A-TCF7L2 positive feedback loop whereby Wnt/β-catenin signaling and m^6^A methylation mutually reinforce each other to promote gastric cancer progression and metastasis, and holds promise for the development of effective diagnosis and treatment strategies against gastric cancer.

## Supplementary information

Supplementary Materials for A WNT7B-m6A-TCF7L2 positive feedback loop promotes gastric cancer progression and metastasis

## References

[1] Liu J, Harada BT, He C (2019). Regulation of gene expression by N(6)-methyladenosine in cancer. Trends Cell Biol..

[2] Wang Q (2020). METTL3-mediated m(6)A modification of HDGF mRNA promotes gastric cancer progression and has prognostic significance. Gut.

[3] Yang XZ (2018). LINC01133 as ceRNA inhibits gastric cancer progression by sponging miR-106a-3p to regulate APC expression and the Wnt/beta-catenin pathway. Mol. Cancer.

[4] Nusse R, Clevers H (2017). Wnt/β-catenin signaling, disease, and emerging therapeutic modalities. Cell.

[5] Yang DD (2020). METTL3 promotes the progression of gastric cancer via targeting the MYC pathway. Front Oncol..

