# Supplementary material for: A WNT7B-m6A-TCF7L2 positive feedback loop promotes gastric cancer progression and metastasis
Source: Signal Transduct Target Ther. 2021 Feb 2;6:43. doi: 10.1038/s41392-020-00397-z (PMC7851143; doi:10.1038/s41392-020-00397-z)
Supplement: Supplementary file 1 — Supplementary Materials for A WNT7B-m6A-TCF7L2 positive feedback loop promotes gastric cancer progression and metastasis [file 41392_2020_397_MOESM1_ESM.docx]

Supplementary Materials for

A WNT7B-m^6^A-TCF7L2 positive feedback loop promotes gastric cancer progression and metastasis

Qian Gao^1,2,†^, Liuyang Yang^1,2,3,^ ^†^, Aolin Shen^3,4^, Yang Li^2^, Yongxiang Li^4^, Shilian Hu^1,3^, Runhuai Yang^5^, Xiangting Wang^1^, Xuebiao Yao^6^* and Guodong Shen^1,3^*

Correspondence to: gdshen@ustc.edu.cn, yaoxb@ustc.edu.cn

**This PDF file includes:**

Materials and Methods

Figures. S1 to S7

Tables S1 to S3

**Materials and Methods**

Patients and Clinical specimens

Patients with histological diagnosis of gastric cancer who have no history of other types of tumors or underlying diseases and received no radiotherapy or chemotherapy before surgical resections were included in this study. Patients who were pregnant or breast-feeding, or have medical or ethical reasons affecting the continuation of the study were excluded. Forty pairs of gastric cancer tissues and matched non-cancerous tissues from the same patient were collected for quantitative reverse transcription-polymerase chain reaction (qRT-PCR) analysis. The matched gastric tissues dissected adjacent to the tumor were collected not less than 3 cm from the tumor margin and used as control group in qRT-PCR analysis to reduce individual-specific and anatomical site-specific effects. Paraffin-embedded gastric tissues from patients with gastric cancer (n = 124) were collected for immunohistochemistry analysis. Gastric tissues from patients with mild chronic gastritis who presented normal gastric mucosa or gastric mucosa with minimal inflammatory changes (n = 30) were histologically evaluated by two independent experts in pathology from the Department of Pathology (The First Affiliated Hospital of USTC, Anhui, China) and constituted the control group in immunohistochemistry analysis. The above specimens and serum samples from 69 gastric cancer patients and 30 healthy people undergoing health check-ups were both obtained from the First Affiliated Hospital of USTC. Overall survival was defined as the interval from tumor resection to death or last follow-up examination; for the analysis of recurrence rate, time to recurrence was defined as the time from the date of resection to the date of recurrence.

Heterotopic, orthotopic and PDX mouse models

BALB/c-nude and NOD/SCID mice (4 weeks old) were purchased from Vital River Laboratory Animal Technology Co., Ltd (Beijing, China) and housed in specific pathogen-free units. To establish heterotopic mouse models, BALB/c-nude mice were injected with gastric cancer cells (3 × 10^6^ cells in 100 μL sterile PBS) subcutaneously in their backs. For orthotopic mouse models, gastric cancer cells (1.5 × 10^6^ cells in 50 μL sterile PBS) were orthotopically inoculated into the middle wall of the greater curvature of the glandular portion of the stomachs of BALB/c-nude mice using a 29-gauge needle. Additionally, fragments from one gastric cancer patient (~2 mm per side) were subcutaneously inoculated into backs of NOD/SCID mice to establish PDX models. WNT7B (100 ng), iCRT-14 (400 μg) or vehicle was injected into one tumor nodule twice a week.

Cell culture, plasmids, and transfection

Human gastric cancer cell lines (SGC7901, NCI-N87, MKN-45, and MGC803) and human gastric epithelia cell line GES-1 were obtained from the Cell Bank of Chinese Academy of Sciences (Shanghai, China). Human gastric cancer cell line 44As3 and HSC44-PE were kindly provided by Prof. Yoshifumi Takei (Aichi Gakuin University, Japan). All cell lines were maintained with RPMI1640 medium (Invitrogen) with 10% fetal bovine serum (Gibco). Two shRNA plasmids targeting human *TCF7L2* and siRNAs against human *METTL3* and *WNT7B* were purchased from GenePharma (Shanghai, China) and the plasmid overexpressing TCF7L2 (GeneCopoeia) was generated by inserting full length cDNA of human *TCF7L2* into pEGFP-puro plasmid. The plasmid overexpressing METTL3 (Hanbio) was generated by inserting full length cDNA of human *METTL3* into pEGFP-puro plasmid. The sequences of shRNAs and siRNAs are shown in Table S3. Lipofectamine 2000-mediated transfection was conducted according to the manufacturer’s instructions (Invitrogen). Stable cell lines were established via puromycin (Invitrogen) selection (2 μg/mL).

Reagents and antibodies

Recombinant human WNT7B (H0007477-P01) was obtained from Abnova (China). All chemicals and iCRT-14 were purchased from Sigma-Aldrich (USA). ELISA kit for human WNT7B protein (SCP546Hu) was from Cloud-Clone Corp (USA). Cell fractionation kit (9038) was from Cell Signaling Technology (USA). The Dual-Luciferase System (E1910) was from Promega (USA). MA2 was kindly provided by Prof. Cai-Guang Yang (Shanghai Institute of Materia Medica, Chinese Academy of Sciences, China).

Antibodies against TCF7L2 (2569), β-actin (3700), TCF1 (2203), TCF3 (2883), LEF1 (2230), Histone H3 (14269), α-tubulin (3875), FTO (31687), β-catenin (8480) were obtained from Cell Signaling Technology. Antibodies against WNT7B (ab155313) were obtained from Abcam (UK). Uncropped and unedited images for all blots have been shown in Fig. S7.

Immunohistochemistry

Paraffin-embedded gastric tissues were processed into 5 μm-thick sections and mounted on slides for staining. The slides were deparaffinised and heat-induced epitope retrieval was performed in citrate buffer (pH 6.0). After quenching endogenous peroxidases, the slides were incubated with blocking serum. Afterwards, the slides were incubated overnight with anti-TCF7L2 antibody, anti-FTO antibody or anti-WNT7B antibody at 4 °C and then with biotin-conjugated secondary antibody and horseradish peroxidase-conjugated streptavidin (Cell Signaling Technology). Binding was detected by DAB substrate kit (Cell Signaling Technology). The slides were then counter stained with hematoxylin. All the slides were scanned using a Leica DM2500 microscope, and images were quantitatively analyzed by Image J software with IHC Toolbox plug-in based on both average staining intensity and frequency of positive cells. Furthermore, the median of immunostaining score values was used as a cut-off to define low versus high group.

For immunhistofluorescence, the slides were stained with anti-TCF7L2 antibody, followed by fluorescent-labeled secondary antibody (Cell Signaling Technology). Nuclei were stained with DAPI. Images were taken with a Zeiss LSM800 confocal laser scanning microscope.

Quantitative reverse transcription-polymerase chain reaction (qRT-PCR)

Total RNA from gastric tissues or gastric cancer cells was isolated using innuPREP RNA Mini kit (Analytik Jena) according to the manufacturer’s protocol. 1 μg of RNA was reverse transcribed into cDNA with TransScript All-in-One First-Strand cDNA Synthesis SuperMix kit (Transgen Biotech). Then quantitative real-time polymerase chain reaction assay was performed using TB Green Premix Ex Taq II (Takara) in technical triplicates with ABI 7500 Sequence Detection System (Applied Biosystems). The value for each sample was calculated from the average of three technical replicates and results were normalized according to internal control *GAPDH* mRNA expression. The sequences of primers are shown in Table S3.

Cell proliferation, migration, invasion, self-renewal assay

The CCK-8 assay was used to evaluate cell proliferation. After transfection, gastric cancer cells were seeded in 96-well plates at a density of 3 × 10^3^ cells/well in 100 μL of complete medium and cultured for 24, 48, 72 and 96 hours. After 2 hours incubation with 10 μL of CCK-8 (Dojindo Molecular Technologies), the optical density was measured at 450 nm.

Cell migration and invasion were analyzed using Boyden chamber assay (96-well insets, 8-μm pore size, Corning). For the migration assay, gastric cancer cells were seeded on the top of non-coated membranes at a density of 5 × 10^3^ cells/well in 100 μL of serum-free medium. For the invasion assay, gastric cancer cells were seeded on the top of Matrigel-coated (Corning) membranes at a density of 1.2 × 10^4^ cells/well in 100 μL of serum-free medium. For both assays, 200 μL complete medium was added to the lower chamber. After culture for 12 hours, cells remaining at the upper surface of the membrane were removed using a swab. Cells that migrated to the lower membrane surface were representative of the migrated or invaded cells. After fixation with 4% paraformaldehyde and staining with 0.1% crystal violet solution, cells were counted.

Soft agar colony formation assay and sphere formation assay were used to analyze cell self-renewal. Soft agar colony formation assay was performed in 6-well plates. Each well was coated with 1.5 mL 0.5% agar (Sigma) mixed with complete medium. Stable transfectants of gastric cancer cells were seeded at a density of 2 × 10^3^ cells/well in a 1.5 mL of 0.35% agar mixed with complete medium. Sphere formation assay was performed in 6-well Ultra Low Attachment plates (Corning). Stable transfectants of gastric cancer cells were seeded at a density of 5 × 10^3^ cells/well in 2 mL serum-free DMEM/F12 supplemented with growth factors EGF, β-FGF, and IGF-1 at a concentration of 20 ng/ml (PeproTech). After cultured for 7 days, both cells were photographed under a microscope. Spheres with a diameter of over 20 μm were counted and analyzed using Image J software.

Human stem cell antibody array

Gastric cancer cells were collected with 5 mM EDTA in PBS and lysed for 1 hour at 4 °C in RIPA buffer containing protease/phosphatase inhibitor (Cell Signaling Technology). After removal of cell debris, protein in cell lysates was quantified using the standard Bradford protein assay. The antibody array (AAH-SC-1, Raybiotech) was performed according to the instructions of the manufacturer. In this array, antibodies against 15 human stem cell biomarkers were spotted in duplicate on nitrocellulose membranes. The human stem cell biomarkers are listed as follows: Alpha-fetoprotein, BMPR-IA, ALK-6, Brachyury, CD38, E-Cadherin, GATA4, hCGbeta, Nanog, Nestin, OCT4, PDX-1, SOX17, SOX2 and VEGFR2. Firstly, cell lysate was incubated with the membrane overnight. Thereafter, the membranes were incubated with a cocktail of biotinylated detection antibodies and then streptavidin-HRP. Finally, membranes were detected using an ECL chemiluminescent system (Thermo). To quantify expression levels, the integrated optical density of each spot was measured using Image J software. The data were corrected for background signal and then normalized to the positive controls on the same membrane.

Proteomic analysis, liquid chromatography coupled with tandem mass spectrometry (LC-MS/MS) analysis, and bioinformatics

Gastric cancer cells were firstly grounded into powder with liquid nitrogen and subsequently sonicated three times on ice in lysis buffer. The remaining debris was removed by centrifugation. Then, the protein was precipitated with cold 15% trichloroacetic acid for 2 hours at -20 °C. After centrifugation, the supernatant was discarded and the remaining precipitate was washed with cold acetone. The protein was dissolved and protein concentration was determined by the 2-D Quant kit (GE Healthcare). After digestion, peptide was desalted and dried by vacuum centrifugation. Tandem-mass-tag (TMT-6 plex) labeling was performed according to the manufacturer’s protocol (Thermo). The samples were then fractionated by high pH reverse-phase HPLC.

The peptides were dissolved in solvent A (0.1% formic acid (FA) in 2% acetonitrile (ACN)) and directly loaded on a reversed-phase pre-column (Acclaim PepMap 100, Thermo Scientific). Then peptide separation was performed with a reversed-phase analytical column (Acclaim PepMap RSLC, Thermo Scientific). The gradient was comprised of an increase from 6% to 22% solvent B (0.1% FA in 98% ACN) for 24 minutes, 22% to 35% in 8 minutes climbing to 80% in 4 minutes then holding at 80% for the last 4 minutes, at a constant flow of 300 nL/min on an EASY-nLC 1000 UPLC system. Afterwards, the resulting peptides were analyzed by Q Exactive Plus hybrid quadrupole-Orbitrap mass spectrometer (Thermo). Next, the peptides were subjected to NSI source followed by tandem mass spectrometry (MS/MS) in Q Exactive Plus (Thermo) coupled online to the UPLC. Peptides were analyzed in the Oribitrap at a resolution of 70,000 and selected for MS/MS using NCE setting as 30, while ion fragments were analyzed in Oribitrap at a resolution of 17,500. A data-dependent procedure which alternated between one MS scan followed by 20 MS/MS scans with 30 seconds dynamic exclusion was applied for the top 20 precursor ions above a threshold ion count of 2E4. Electrospray voltage of 2 KV was applied. Automatic gain control was used for preventing overfilling of ion trap and 5E4 ions were accumulated to generate MS/MS spectra. The m/z scan range was 350 to 1800. Fixed first mass was set as 100 m/z.

The resulting MS/MS data were analyzed with Mascot search engine. For protein quantification, TMT-6-plex was selected in Mascot; false discovery rate was adjusted to < 1% and peptide ion score was set ≥ 20. Then protein quantification was performed using the median values of all peptide spectral matches of the protein group. Four biological replicates were performed. Pathway analysis of differential protein expression was obtained from the Kyoto Encyclopedia of Genes and Genomes (KEGG) database. Briefly, the KEGG database description of differentially expressed proteins was annotated using the KAAS online tools and then mapped on the KEGG pathway database using KEGG online service tools KEGG mapper.

RT^2^ profiler PCR array

The human Wnt signaling pathway RT^2^ Profiler PCR arrays were carried out according to manufacturer’s instructions (QIAGEN). Total RNA extraction and purification from gastric cancer cells were performed using the RNeasy Elute kit (Invitrogen). After DNase enzyme digestion to exclude genomic DNA contamination, RNA was quantified with a NanoDrop spectrophotometer (Thermo). RNA (1 μg) was reverse transcribed using a cDNA conversation kit (QIAGEN). The cDNA was used in the real-time RT^2^ Profiler PCR Array (PAHS-043Y) consisting of 84 genes involved in Wnt signaling pathway as well as five housekeeping genes. Data were analyzed using the online analysis software (RT^2^ Profiler PCR Array Data analysis version 3.5). Gene expression was normalized to housekeeping genes. Three biological replicates were analyzed for each experimental condition. Mean values of fold changes from three biological replicates were calculated and submitted to statistical analysis. A two-fold or greater change in expression was considered significant. Fold-change values greater than one were indicative of an up-regulation, and the fold-regulation is equal to the fold-change. Fold-change values less than one were indicative of down-regulation of the gene expression and the fold-regulation is the negative inverse of the fold-change.

Chromatin Immunoprecipitation (ChIP)

The ChIP assay was performed using SimpleChIP Assay Kit (Cell Signaling Technology) according to the manufacturer’s instructions. Briefly, N87 cells were first cross-linked with 1% formaldehyde for 10 minutes. Then glycine was added to a final concentration of 0.125 M, and the incubation was continued for another 5 minutes. Afterwards, cells were collected and lysed. After sonication and removal of a control input, lysate was incubated with anti-TCF7L2 antibody or control rabbit IgG at 4 °C overnight. Then protein A-agarose beads pre-blocked with salmon sperm DNA were added and the incubation was continued for another 2 hours. After washing the beads, immunocomplexes were eluted, followed by treatment with RNase A and proteinase K. The purified DNA was analyzed by real-time PCR using locus specific primers and normalized to input DNA. Four sets of primers were designed to cover the putative binding sites predicted using the JASPAR database, and listed in Table S3.

Luciferase reporter assay

The transcriptional activity of Wnt/β-catenin signaling was detected by the dual-luciferase assay using TOP^Flash^ reporter. Briefly, gastric cancer cells (1 × 10^4^) were transfected with 200 ng of either TOP^Flash^ or FOP^Flash^ with 10 ng of pRL-TK plasmid (Promega). After 24 hours of transfection, cells were treated with WNT7B or LiCl for 24 hours. Then both firefly and renilla luciferase activities were measured in a Glomax multi-detection system luminometer using the Dual-Luciferase Reporter Assay System (Promega). Firefly luciferase activity was normalized for transfection efficiency against renilla luciferase activity. The luciferase activity was normalized against those of cells transfected with FOP^Flash^ plasmid.

The sequence of WNT7B is located at chromosome 22, NC 00022.11 (45920362. .45977162, complement) in the human genome. The *WNT7B* promoter-luciferase reporter plasmid was constructed by inserting 2-kb region upstream of transcript start site into pGL3-Basic reporter plasmid. *WNT7B* promoter activity was analyzed by luciferase assay with the *WNT7B* promoter-luciferase reporter plasmid.

m^6^A-RNA immunoprecipitation (RIP)-qPCR

Real-time PCR was performed to assess the relative abundance of *TCF7L2* mRNA in antibody-immunoprecipitated samples and input samples. Briefly, total RNA was isolated with innuPREP RNA Mini kit (Analytik Jena) and then fragmented. 500 ng of RNA was kept as an input sample, while the remaining RNA was immunoprecipitated with control IgG or anti-m^6^A antibody (202003, Synaptic Systems). The immunoprecipitated RNA was washed, eluted and concentrated with an RNA Clean and Concentrator-5 kit (Zymo Research). The same amount of the immunoprecipitated RNA or input RNA from each sample was reverse transcribed into cDNA with TransScript All-in-One First-Strand cDNA Synthesis SuperMix kit (Transgen Biotech) and analyzed by real-time PCR. *HPRT1*, a cellular mRNA containing no m^6^A site, served as a negative control. The corresponding m^6^A enrichment in each sample was calculated by normalizing to the input. The primers were designed for five high confidence m^6^A sites of *TCF7L2* mRNA predicted by SRAMP and listed in Table S3.

RNA methylation assay

Global mRNA m^6^A levels were quantified with an ELISA-based EpiQuik™ m6A RNA Methylation Quantification Kit (P-9005, Epigentek) according to the manufacturer’s instructions. Briefly, 200 ng of poly-A-purified RNA from gastric cancer cells was used for each assay well. An RNA containing no m^6^A served as a negative control, while m^6^A oligos that were normalized to have 100% m^6^A served as a positive control. Capture antibodies and detection antibodies were then added to each well. The abundance of m^6^A in mRNA was quantified colorimetrically by absorbance and then calculated based on the standard curve.

Ethics approval and consent to participate

The animal study was conducted with approval from the Anhui Medical University Institutional Animal Care and Use Committee (LLSC20180351) and was performed in accordance with established guidelines. All clinical samples were collected with written consent form prior to enrollment. The use of clinical samples was evaluated and approved by the clinical research review board of The First Affiliated Hospital of USTC (2019-XH-001). The experimental methods complied with the Helsinki Declaration.

Data availability

All data and materials are available from the corresponding authors upon reasonable request.

Statistical analysis

All data were collected from more than three independent experiments, presented as the mean ± standard error of the mean and analyzed using SPSS 16.0 software. The specific statistical test performed for each data has been included in the figure legends. *P* values less than 0.05 were considered statistically significant.

Figure. S1.


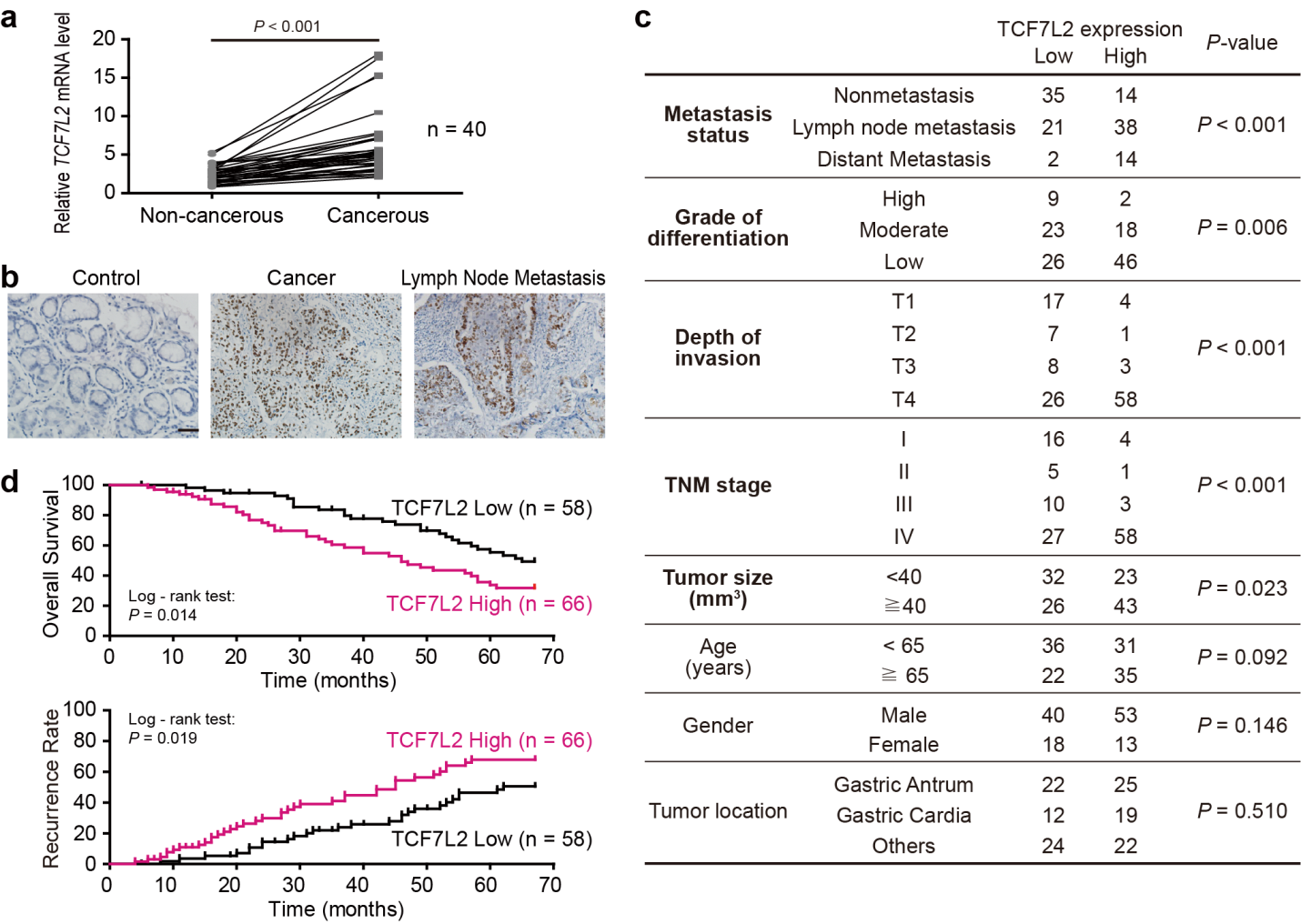


**Figure. S1.** Increased expression of TCF7L2 in gastric cancer tissues is associated with aggressive clinical features and poor prognosis.

**a** Levels of *TCF7L2* mRNA in cancerous tissues and non-cancerous tissues from the same patients with gastric cancer were analyzed by qRT-PCR. *P* values were calculated by the paired Student *t* test. **b** Immunohistochemical analysis of TCF7L2 protein in gastric tissues from patients with gastric cancer (Cancer) and mild chronic gastritis (Control). Scale bars = 50 μm. **c** Correlation between TCF7L2 expression and the clinicopathological features in 124 patients with gastric cancer. *P* values were determined by the two-sided Fisher exact test. **d** Survival curves and recurrence rates of gastric cancer patients stratified according to expression of TCF7L2 protein. *P* values were determined by the Kaplan-Meier log-rank test.

Figure. S2.


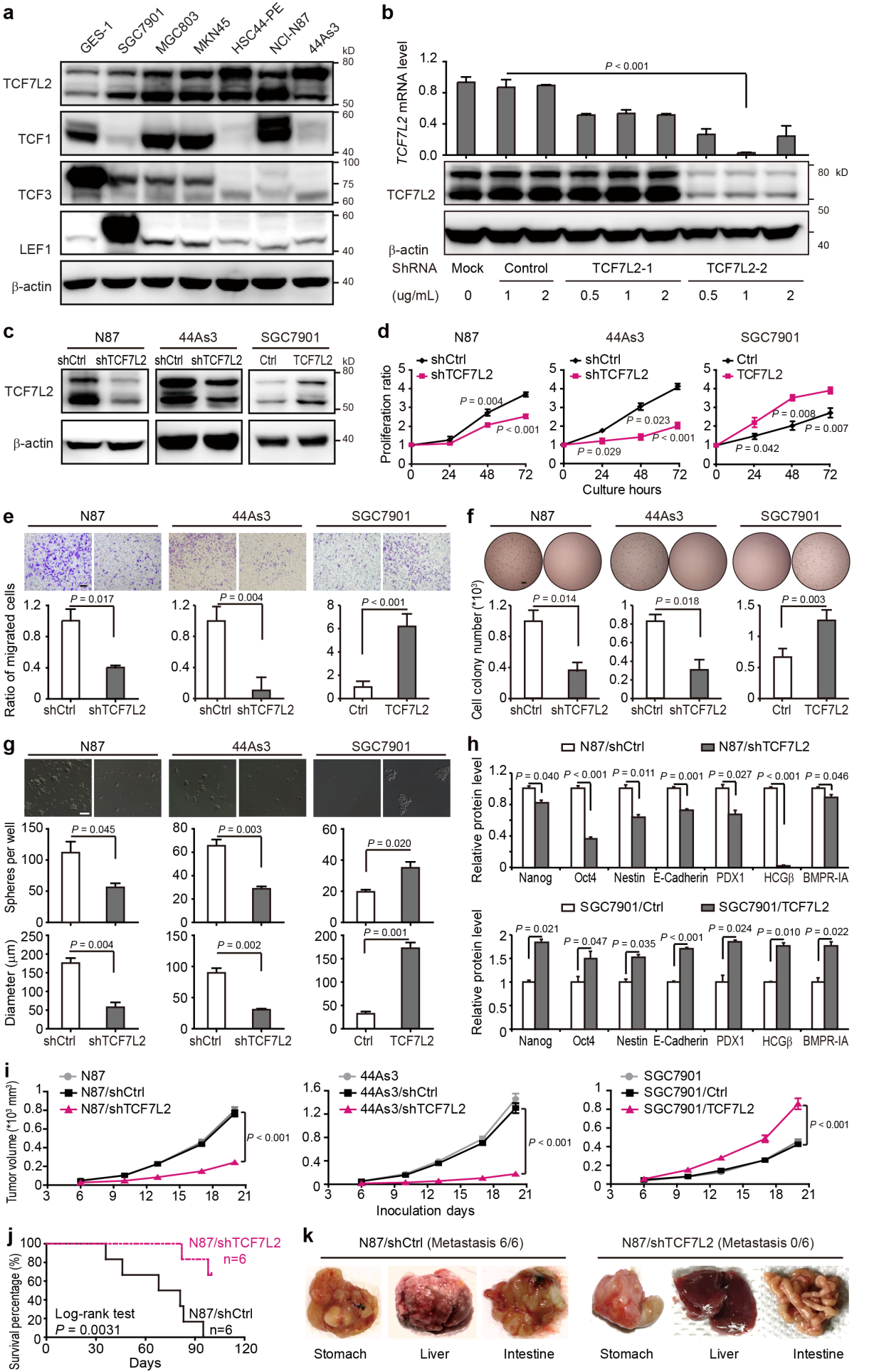


**Figure. S2.** TCF7L2 promotes the malignant phenotype of gastric cancer cells and their ability to metastasize in mice.

**a** Expression of TCF/LEF family members in six gastric cancer cell lines and a normal cell line GES-1 was analyzed by immunoblotting. β-actin was included as a loading control. **b** Levels of *TCF7L2* mRNA and protein in N87 cells after transfection without DNA (Mock), with control shRNA (Control) and specific shRNAs targeting TCF7L2 were analyzed. β-actin is included as a loading control. According to the depletion efficiency, shRNA TCF7L2-2 was used in subsequent experiments. **c** Immunoblotting of TCF7L2 protein in N87 and 44As3 cells after transfection with control shRNA (shCtrl) and TCF7L2 shRNA (shTCF7L2), and SGC7901 cells after transfection with empty plasmid (Ctrl) and TCF7L2 expression plasmid (TCF7L2). β-actin is included as a loading control. **d** Cell proliferation of N87 and 44As3 cells transfected with TCF7L2 shRNA, and SGC7901 cells transfected with TCF7L2 plasmid were measured by CCK8 assay at different time points. **e** Transwell analysis of N87 and 44As3 cells transfected with TCF7L2 shRNA, and SGC7901 cells transfected with TCF7L2 plasmid. The upper panel presents representative images. Scale bars = 200 μm. The lower panel shows ratio of migrated cells. **f** Soft agar colony formation analysis of N87 and 44As3 cells stably transfected with TCF7L2 shRNA, and SGC7901 cells stably transfected with TCF7L2 plasmid. The upper panel presents representative images. Scale bars = 500 μm. The lower panel shows number of cell colonies. **g** Sphere formation analysis of N87 and 44As3 cells stably transfected with TCF7L2 shRNA, and SGC7901 cells stably transfected with TCF7L2 plasmid. The upper panel presents representative images. Scale bars = 100 μm. The middle and lower panels show number and diameter of spheres, respectively. **h** Expression of stem cell markers in N87 cells transfected with TCF7L2 shRNA and SGC7901 cells transfected with TCF7L2 plasmid was analyzed by antibody arrays and quantitatively measured using Image-Pro Plus 6.0 software. All *P* values were calculated by the unpaired Student *t* test. **i** Mean tumor volumes were measured at indicated time points after mice heterotopic injection with stable transfectants or untreated gastric cancer cells. N = 8 mice per group; *P* values were calculated by two-way repeated measure ANOVA with post-hoc LSD test. **j** Kaplan-Meier survival curves of mice orthotopically injected with stable N87/shCtrl or stable N87/shTCF7L2 cells. *P* values were calculated by log-rank test. **k** Incidence and representative images of tumor metastasis in mice orthotopically injected with N87/shCtrl or N87/shTCF7L2 cells.

Figure. S3.


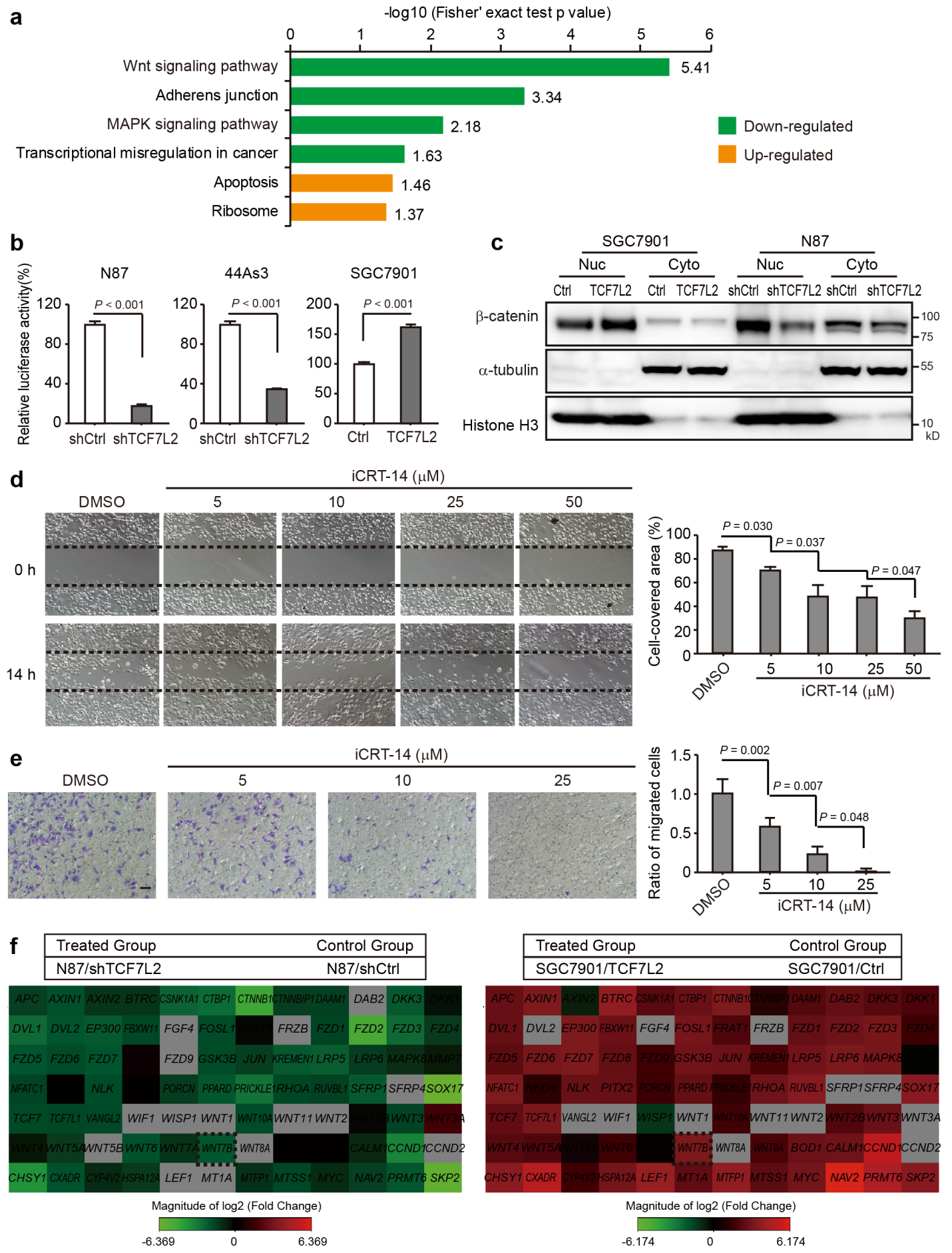


**Figure. S3.** TCF7L2 enhances the expression of Wnt/β-catenin target genes.

**a** KEGG pathway functional enrichment of differentially expressed proteins in N87 cells transfected with TCF7L2 shRNA. **b** TOP^Flash^ luciferase reporter assay in N87 and 44As3 cells transfected with TCF7L2 shRNA, and SGC7901 cells transfected with TCF7L2 plasmid. *P* values were calculated by unpaired t test. **c** Nuclear (Nuc) and cytoplasmic (Cyto) levels of β-catenin in N87 cells transfected with TCF7L2 shRNA and SGC7901 cells transfected with TCF7L2 plasmid were analyzed by immunoblotting. Histone H3 and α-tubulin were used for loading controls and markers for nuclear and cytoplasmic fractions, respectively. **d** Wound healing analysis of N87 cells in the presence of iCRT-14. The left panel shows representative images. Horizontal lines indicate the wound edge. Scale bars = 100 μm. The right panel presents the percentage of cell-covered area which was quantitatively measured by Image-Pro Plus 6.0 software. *P* values were calculated by one-way ANOVA with post-hoc LSD test. **e** Transwell analysis of N87 cells in presence of iCRT-14. The left panel presents representative images. Scale bars = 100 μm. The right panel shows the ratio of migrated cells. *P* values were calculated by one-way ANOVA with post-hoc LSD test. **f** Heatmap illustrated changes in mRNA expression of Wnt/β-catenin signaling-related genes in N87 cells transfected with TCF7L2 shRNA and SGC7901 cells transfected with TCF7L2 plasmid.

Figure. S4.


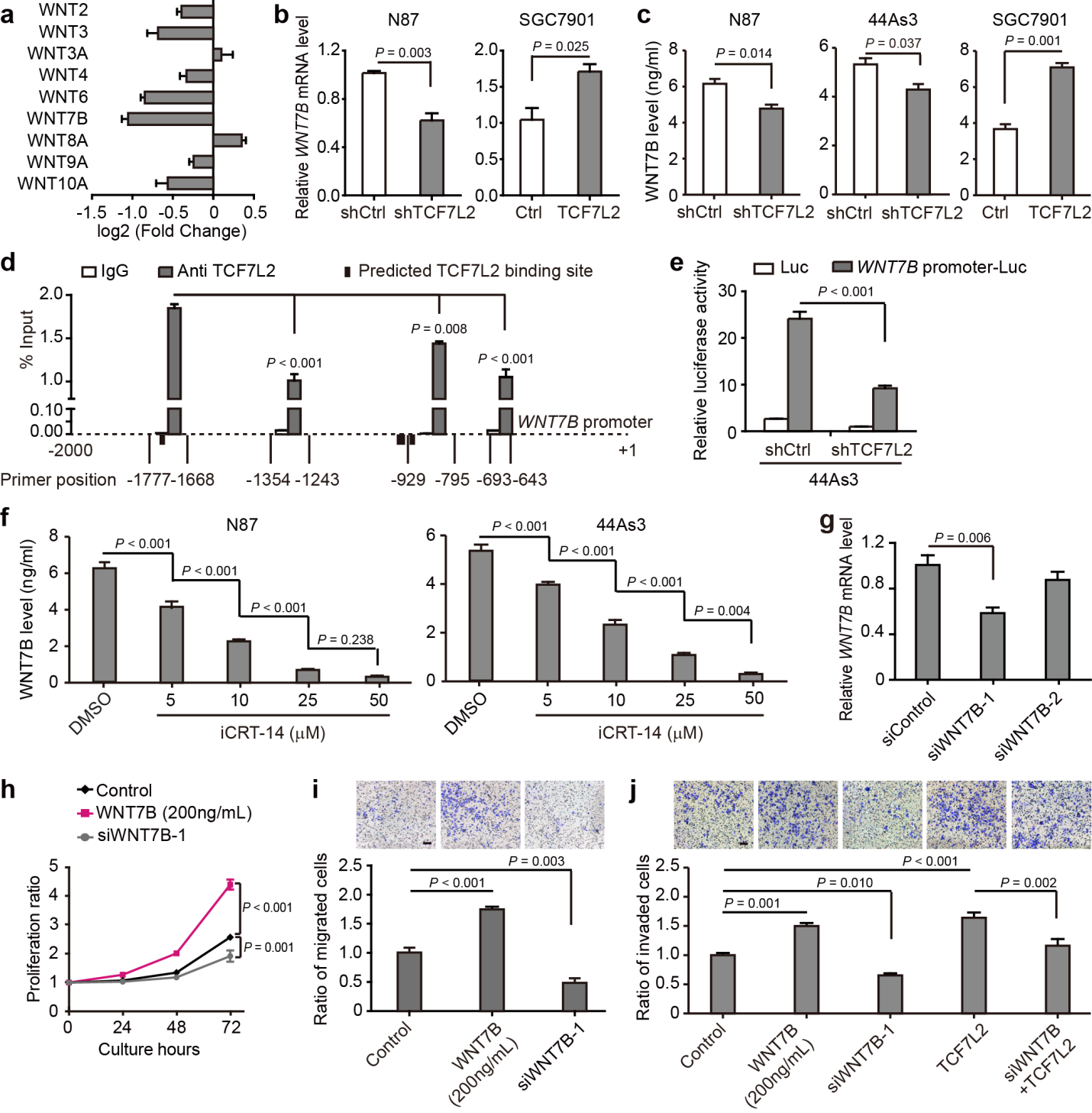


**Figure. S4.** TCF7L2 triggers WNT7B expression in gastric cancer.

**a** The protein fold changes of WNTs following TCF7L2 knockdown were detected by quantitative proteomic analysis. **b** Levels of *WNT7B* mRNA in N87 cells transfected with TCF7L2 shRNA and SGC7901 cells transfected with TCF7L2 plasmid were analyzed by qRT-PCR. *P* values were calculated with the unpaired Student *t* test. **c** Levels of WNT7B protein in the cell culture medium of N87 and 44As3 cells transfected with TCF7L2 shRNA and SGC7901 cells transfected with TCF7L2 plasmid were analyzed by enzyme-linked immunosorbent assay (ELISA). *P* values were calculated with the unpaired Student *t* test. **d** The recruitment of TCF7L2 to the *WNT7B* promoter was analyzed by ChIP assay. Four sets of primers were designed around each putative TCF7L2-binding site. Nucleotide positions are numbered with respect to the transcription start site (+1). *P* values were calculated with one-way ANOVA with post-hoc LSD test. **e** Luciferase reporter assay for *WNT7B* promoter in 44As3 cells transfected with TCF7L2 shRNA. *P* values were calculated by the unpaired Student *t* test. **f** Levels of WNT7B protein in the cell culture medium of N87 and 44As3 cells treated with iCRT-14 for 24 hours were analyzed by ELISA. *P* values were calculated by one-way ANOVA with post-hoc LSD test. **g** Levels of WNT7B mRNA in N87 cells after transfection with control siRNA (Control) and specific siRNAs targeting WNT7B were analyzed by qRT-PCR. *P* values were calculated by one-way ANOVA with post-hoc LSD test. **h** Cell proliferation of N87 cells transfected with WNT7B siRNA or treated with WNT7B protein were measured by CCK8 assay at different time points. *P* values were calculated by two-way ANOVA with post-hoc LSD test. **i** Transwell analysis of N87 cells transfected with WNT7B siRNA or treated with WNT7B protein. The upper panel presents representative images. Scale bars = 200 μm. The lower panel shows ratio of migrated cells. *P* values were calculated by one-way ANOVA with post-hoc LSD test. **j** Cell invasion of N87 cells treated with WNT7B protein, or transfected with WNT7B siRNA, TCF7L2 plasmid or both together. The upper panel presents representative images. Scale bars = 200 μm. The lower panel shows ratio of invaded cells. *P* values were calculated by one-way ANOVA with post-hoc LSD test.

Figure. S5.


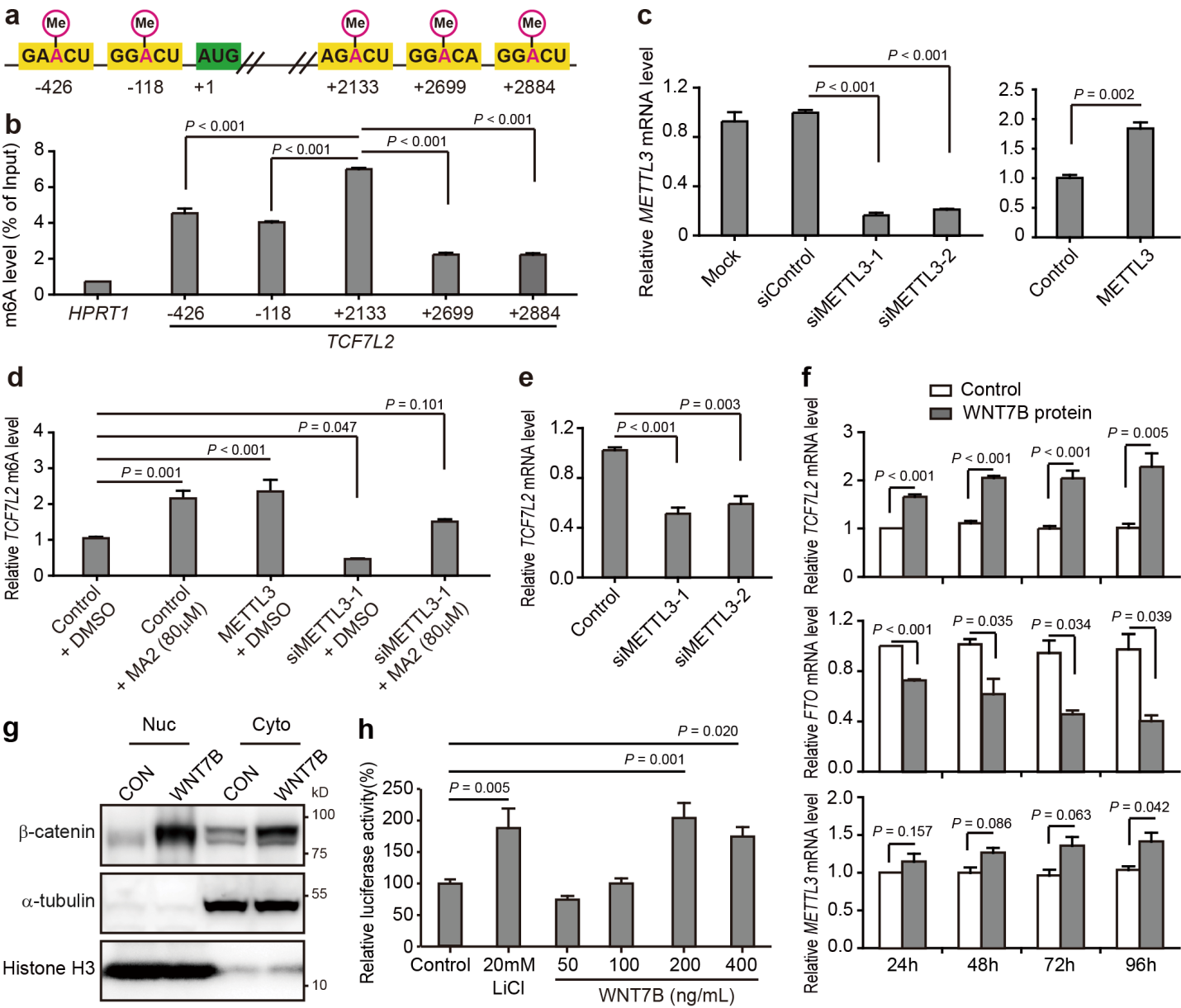


**Figure. S5.** WNT7B promotes TCF7L2 expression by up-regulating m^6^A mRNA methylation.

**a** Schematic indicates the location of five high confidence m^6^A sites predicted by SRAMP in *TCF7L2* mRNA. Nucleotide positions are numbered with respect to the transcription start site (+1). **b** Gene-specific m^6^A-RIP-qPCR analysis of predicted m^6^A sites. *HPRT1* was included a negative control. **c** Levels of *METTL3* mRNA in N87 cells after transfection without RNA (Mock), with control siRNA (siControl), specific siRNAs targeting METTL3, control plasmid (Control), or METTL3 plasmid were analyzed by qRT-PCR. *P* values were calculated by one-way ANOVA with post-hoc LSD test (left panel) and unpaired Student *t* test (right panel). **d** m^6^A methylation at site +2133 of *TCF7L2* mRNA in N87 cells treated with MA2, transfected with METTL3 plasmid, METTL3 siRNA, or transfected with METTL3 siRNA in presence of MA2 was analyzed by gene-specific m^6^A-RIP-qPCR. *P* values were calculated by one-way ANOVA with post-hoc LSD test. **e** Levels of *TCF7L2* mRNA in N87 cells transfected with METTL3 siRNAs were analyzed by qRT-PCR. **f** mRNA levels of *TCF7L2*, *FTO* and *METTL3* at indicated time points were analyzed by qRT-PCR in N87 cells treated with WNT7B protein (200 ng/mL). *P* values were calculated with the unpaired Student *t* test. **g** Nuclear (Nuc) and cytoplasmic (Cyto) levels of β-catenin in N87 cells treated with WNT7B protein (200 ng/mL) for 48 hours were analyzed by immunoblotting. Histone H3 and α-tubulin were used as loading controls and markers for nuclear and cytoplasmic fractions, respectively. **h** TOP^Flash^ luciferase reporter assay in N87 cells treated with LiCl or WNT7B for 24 hours. LiCl served as a positive control. *P* values were calculated by one-way ANOVA with post-hoc LSD test.

Figure. S6.


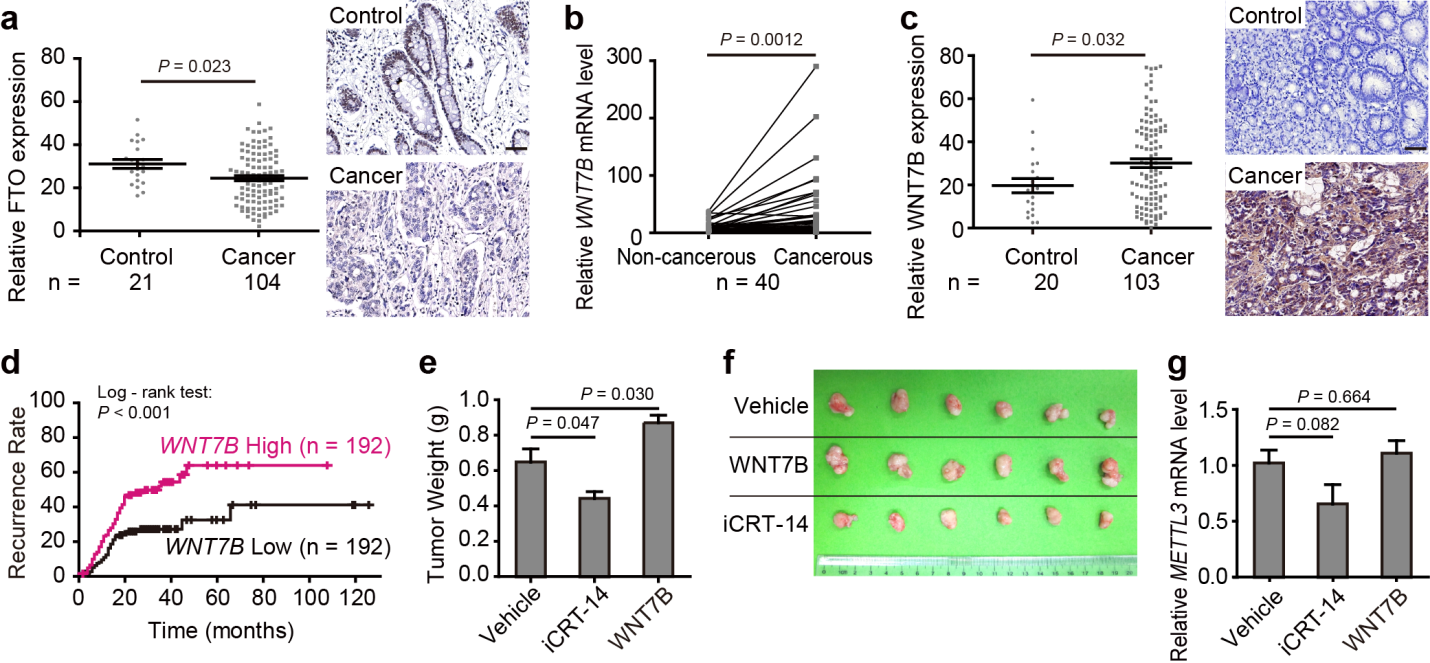


**Figure. S6.** Clinical relevance and therapeutic targeting of WNT7B-m^6^A-TCF7L2 positive feedback loop in gastric cancer.

**a** Immunohistochemical analysis of FTO protein in gastric tissues from patients with gastric cancer (Cancer) and mild chronic gastritis (Control). The left panel shows quantitative immunostaining data that were measured by Image J software. *P* values were calculated with the unpaired Student *t* test. The right panel presents representative images. Scale bars = 50 μm. **b** Levels of *WNT7B* mRNA in cancerous tissues and non-cancerous tissues from the same patients with gastric cancer were analyzed by qRT-PCR. *P* values were calculated with the paired Student *t* test. **c** Immunohistochemical analysis of WNT7B protein in gastric tissues from patients with gastric cancer (Cancer) and mild chronic gastritis (Control). The left panel shows quantitative immunostaining data that were measured by Image J software. *P* values were calculated with the unpaired Student *t* test. The right panel presents representative images. Scale bars = 50 μm. **d** Recurrence rates of gastric cancer patients stratified according to levels of *WNT7B* mRNA were analyzed using the GEPIA online tool based on TCGA data. *P* values were determined by Kaplan-Meier log-rank test. **e, f** Weight and image of PDX tumors from vehicle, iCRT-14 and WNT7B-treated mice are shown. *P* values were calculated by one-way ANOVA with post-hoc LSD test. **g** mRNA levels of *METTL3* in PDX tumors from vehicle, iCRT-14 and WNT7B-treated mice were analyzed by qRT-PCR. *P* values were calculated by one-way ANOVA with post-hoc LSD test.

Figure. S7.


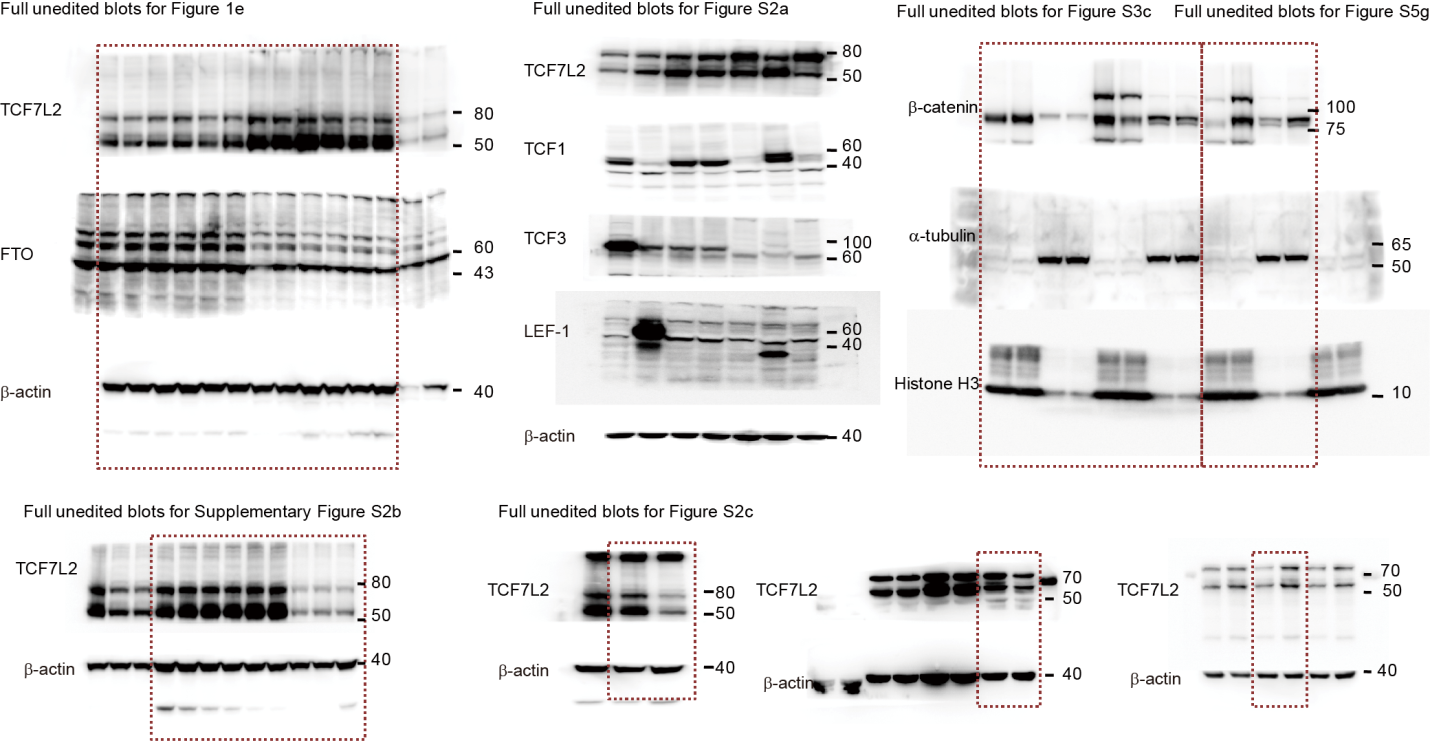


**Figure. S7.** Uncropped and unedited images for all blots.

Table S1.

**Table S1.** All Wnt signaling-related genes with significantly changed expression in N87/shTCF7L2 compared to N87/CON.

^a^ 2-fold changes in expression levels were utilized as cutoff levels.

^b^ *P* values were determined by unpaired Student *t* test. A *P* value cutoff of 0.05 was used to determine significant differential expression.

| **Gene Symbol** | **Fold Regulation^a^** | ***P*-Value^b^** |
| --- | --- | --- |
| APC | -3.17 | 0.000000 |
| AXIN1 | -4.35 | 0.000000 |
| AXIN2 | -2.59 | 0.000000 |
| BTRC | -2.64 | 0.000000 |
| CSNK1A1 | -6.15 | 0.000000 |
| CTBP1 | -7.20 | 0.000000 |
| CTNNB1 | -30.43 | 0.000000 |
| CTNNBIP1 | -3.01 | 0.000000 |
| DAAM1 | -3.29 | 0.000000 |
| DKK1 | -5.11 | 0.000000 |
| DVL1 | -7.01 | 0.000000 |
| DVL2 | -4.57 | 0.000000 |
| EP300 | -5.14 | 0.000000 |
| FBXW11 | -3.62 | 0.000000 |
| FOSL1 | -3.91 | 0.000000 |
| FZD1 | -3.35 | 0.000000 |
| FZD2 | -39.95 | 0.000000 |
| FZD3 | -7.07 | 0.000000 |
| FZD4 | -2.86 | 0.000000 |
| FZD5 | -4.58 | 0.000000 |
| FZD6 | -3.88 | 0.000000 |
| FZD7 | -4.64 | 0.000000 |
| GSK3B | -3.91 | 0.000000 |
| JUN | -3.02 | 0.000000 |
| KREMEN1 | -5.15 | 0.000000 |
| LRP5 | -5.45 | 0.000000 |
| LRP6 | -2.90 | 0.000000 |
| MAPK8 | -2.30 | 0.000000 |
| NFATC1 | -3.32 | 0.000000 |
| NLK | -3.82 | 0.000000 |
| PORCN | -2.90 | 0.000000 |
| PPARD | -4.70 | 0.000000 |
| PRICKLE1 | -8.04 | 0.000000 |
| RHOA | -3.59 | 0.000000 |
| RUVBL1 | -3.19 | 0.000000 |
| SFRP1 | -5.02 | 0.000000 |
| SOX17 | -82.67 | 0.000000 |
| TCF7 | -3.68 | 0.000000 |
| TCF7L1 | -3.57 | 0.000000 |
| VANGL2 | -3.78 | 0.000000 |
| WNT10A | -3.83 | 0.000000 |
| WNT3 | -3.39 | 0.000000 |
| WNT3A | 3.35 | 0.000000 |
| WNT5A | -2.80 | 0.000000 |
| WNT6 | -4.43 | 0.000000 |
| WNT7A | -2.56 | 0.000000 |
| WNT7B | -4.94 | 0.000000 |
| CALM1 | -2.08 | 0.000000 |
| CCND1 | -11.74 | 0.000000 |
| CHSY1 | -16.35 | 0.000000 |
| CXADR | -4.37 | 0.000000 |
| CYP4V2 | -2.17 | 0.000000 |
| HSPA12A | -2.79 | 0.000000 |
| MTFP1 | -2.42 | 0.000000 |
| MTSS1 | -3.55 | 0.000000 |
| MYC | -2.19 | 0.000000 |
| NAV2 | -3.12 | 0.000000 |
| PRMT6 | -4.70 | 0.000000 |
| SKP2 | -71.17 | 0.000000 |

Table S2.

**Table S2.** All Wnt signaling-related genes with significantly changed expression in SGC7901/TCF7L2 compared to SGC7901/CON.

^a^ 2-fold changes in expression levels were utilized as cutoff levels.

^b^ *P* values were determined by unpaired Student *t* test. A *P* value cutoff of 0.05 was used to determine significant differential expression.

| **Gene Symbol** | **Fold Regulation^a^** | ***P*-Value^b^** |
| --- | --- | --- |
| APC | 6.42 | 0.000000 |
| AXIN1 | 10.56 | 0.000000 |
| BTRC | 15.29 | 0.000000 |
| CSNK1A1 | 5.08 | 0.000000 |
| CTBP1 | 6.73 | 0.000000 |
| CTNNB1 | 9.98 | 0.000000 |
| CTNNBIP1 | 2.09 | 0.000000 |
| DAAM1 | 4.50 | 0.000000 |
| DAB2 | 6.22 | 0.000000 |
| DKK1 | 4.86 | 0.000000 |
| DKK3 | 3.85 | 0.000000 |
| DVL1 | 6.33 | 0.000000 |
| DVL2 | -2.16 | 0.000000 |
| EP300 | 10.26 | 0.000000 |
| FBXW11 | 6.95 | 0.000000 |
| FGF4 | -2.16 | 0.000000 |
| FOSL1 | 8.54 | 0.000000 |
| FRAT1 | 4.06 | 0.000000 |
| FRZB | -2.16 | 0.000000 |
| FZD1 | 6.16 | 0.000000 |
| FZD2 | 7.57 | 0.000000 |
| FZD3 | 9.10 | 0.000000 |
| FZD4 | 2.94 | 0.000000 |
| FZD5 | 5.26 | 0.000000 |
| FZD6 | 8.83 | 0.000000 |
| FZD7 | 10.43 | 0.000000 |
| FZD8 | 6.10 | 0.000000 |
| FZD9 | 2.99 | 0.000000 |
| GSK3B | 8.08 | 0.000000 |
| JUN | 6.93 | 0.000000 |
| KREMEN1 | 3.66 | 0.000000 |
| LRP5 | 8.09 | 0.000000 |
| LRP6 | 9.76 | 0.000000 |
| MAPK8 | 8.45 | 0.000000 |
| NFATC1 | 10.02 | 0.000000 |
| NKD1 | 2.21 | 0.000000 |
| NLK | 6.56 | 0.000000 |
| PITX2 | 6.32 | 0.000000 |
| PORCN | 2.95 | 0.000000 |
| PPARD | 5.83 | 0.000000 |
| PRICKLE1 | 2.59 | 0.000000 |
| RHOA | 7.56 | 0.000000 |
| RUVBL1 | 11.89 | 0.000000 |
| SFRP1 | -2.16 | 0.000000 |
| SFRP4 | -2.16 | 0.000000 |
| SOX17 | 12.32 | 0.000000 |
| TCF7 | 5.71 | 0.000000 |
| TCF7L1 | 9.61 | 0.000000 |
| VANGL2 | -2.16 | 0.000000 |
| WIF1 | -2.16 | 0.000000 |
| WISP1 | -2.54 | 0.000000 |
| WNT1 | -2.16 | 0.000000 |
| WNT10A | 3.17 | 0.000000 |
| WNT11 | -2.16 | 0.000000 |
| WNT2 | -2.16 | 0.000000 |
| WNT2B | 4.25 | 0.000000 |
| WNT3 | 6.75 | 0.000000 |
| WNT3A | -2.16 | 0.000000 |
| WNT4 | 4.79 | 0.000000 |
| WNT5A | 4.10 | 0.000000 |
| WNT6 | 3.00 | 0.000000 |
| WNT7B | 9.69 | 0.000000 |
| WNT8A | -2.16 | 0.000000 |
| WNT9A | 3.69 | 0.000000 |
| BOD1 | 7.25 | 0.000000 |
| CALM1 | 7.52 | 0.000000 |
| CCND1 | 38.30 | 0.000000 |
| CCND2 | -2.16 | 0.000000 |
| CHSY1 | 13.29 | 0.000000 |
| CXADR | 32.80 | 0.000000 |
| CYP4V2 | 3.07 | 0.000000 |
| HSPA12A | 8.50 | 0.000000 |
| LEF1 | 16.50 | 0.000000 |
| MT1A | 5.72 | 0.000000 |
| MTFP1 | 14.88 | 0.000000 |
| MTSS1 | 5.33 | 0.000000 |
| MYC | 8.36 | 0.000000 |
| NAV2 | 72.23 | 0.000000 |
| PRMT6 | 25.73 | 0.000000 |
| SKP2 | 12.26 | 0.000000 |

Table S3.

**Table S3.** shRNAs, siRNAs and Primers.

|  | **Gene** | **Sequence 5’–3’** |
| --- | --- | --- |
| shRNA | | |
|  | TCF7L2-1 | Sense: GGGAUUUAGCUGAUGUCAATT |
|  | TCF7L2-2 | Sense: GCGACAGCUUCAUAUGCAATT |
| siRNA | | |
|  | METTL3-1 | Sense: GCAGAACAGGACUCGACUATT |
|  | METTL3-2 | Sense: GCACUUGGAUCUACGGAAUTT |
|  | WNT7B-1 | Sense: UCAUGAACCUGCAUAACAATT |
|  | WNT7B-2 | Sense: GCAGGGCUACUACAACCAATT |
| Primers for qRT-PCR | | |
|  | TCF7L2 | F: TGGAGGGCTCTTTAAGGGG |
|  |  | R: GATCCGTTGGGGAGGTAGG |
|  | WNT7B | F: CACCTTCCTGCGCATCAAAC |
|  |  | R: GTCCTCCTCGCAGTAGTTGG |
|  | FTO | F: GCCGCTGCTTGTGAGACCTTC |
|  |  | R: TGCTGCTCTGCTCTTAATGTCCAC |
|  | METTL3 | F: ACACTGCTTGGTTGGTGTCA |
|  |  | R: AATCTTTCGAGTGCCAGGGG |
|  | GAPDH | F: CTCGCTCCTGGAAGATGGTGAT |
|  |  | R: CTTCATTGACCTCAACTACATGG |
| Primers for ChIP qPCR | | |
|  | WNT7B (-1777~-1668) | F: GGTTCCCCTGTGTGTGCTGA |
|  |  | R: ATTCCTGGGCCCCTCGAT |
|  | WNT7B (-1354~-1243) | F: TCTGGGTCTTGCCCACTGA |
|  |  | R: CAAAGCCCATCTAGCCTGATT |
|  | WNT7B (-929~-795) | F: GAATCTCCCATTGAGGTTTGCT |
|  |  | R: GAGAGACCACCCACTCCCATT |
|  | WNT7B (-693~-643) | F: GCGCAGGTGTCTCTTCCGA |
|  |  | R: GCTTGGGTGACGAGTGGTG |
| Primers for m^6^A RIP qPCR | | |
|  | HPRT1 | F: TGACACTGGCAAAACAATGCA |
|  |  | R: GGTCCTTTTCACCAGCAAGCT |
|  | TCF7L2 (-426) | F: GCTCCCAGACTACTCCGTTCC |
|  |  | R: GGGAAGCCGAAGATACAGGAG |
|  | TCF7L2 (-118) | F: ACCTTGGACTCGTCTTTTTCTTG |
|  |  | R: TTTTCACCCACCAGCAGCAA |
|  | TCF7L2 (+2133) | F: CCATTCTTATTTCAATTTCTCCTT |
|  |  | R: ATTGGTTCGCAAGCTCGTATT |
|  | TCF7L2 (+2699) | F: GAAACCCAGATGTCACCAAAT |
|  |  | R: TGCTCAGACAGTGTCGCTAAAA |
|  | TCF7L2 (+2884) | F: CAGTGGGAACCATCTTCGTTT |
|  |  | R: TTGGCACGTAAAGTTTTGTACAC |
